# *BCR::ABL1*‐Positive Acute Myeloid Leukemia

**DOI:** 10.1002/ajh.27633

**Published:** 2025-02-11

**Authors:** Alban Canali, Jean‐Baptiste Rieu, Leopoldine Lapierre, Barbara J. Bain

**Affiliations:** ^1^ Haematology Laboratory Cancer University Institute of Toulouse–Oncopole, University Hospital of Toulouse Toulouse France; ^2^ Department of Haematology Cancer University Institute of Toulouse–Oncopole, University Hospital of Toulouse Toulouse France; ^3^ Centre for Haematology, St Mary's Hospital Campus of Imperial College Faculty of Medicine, St Mary's Hospital London UK

1

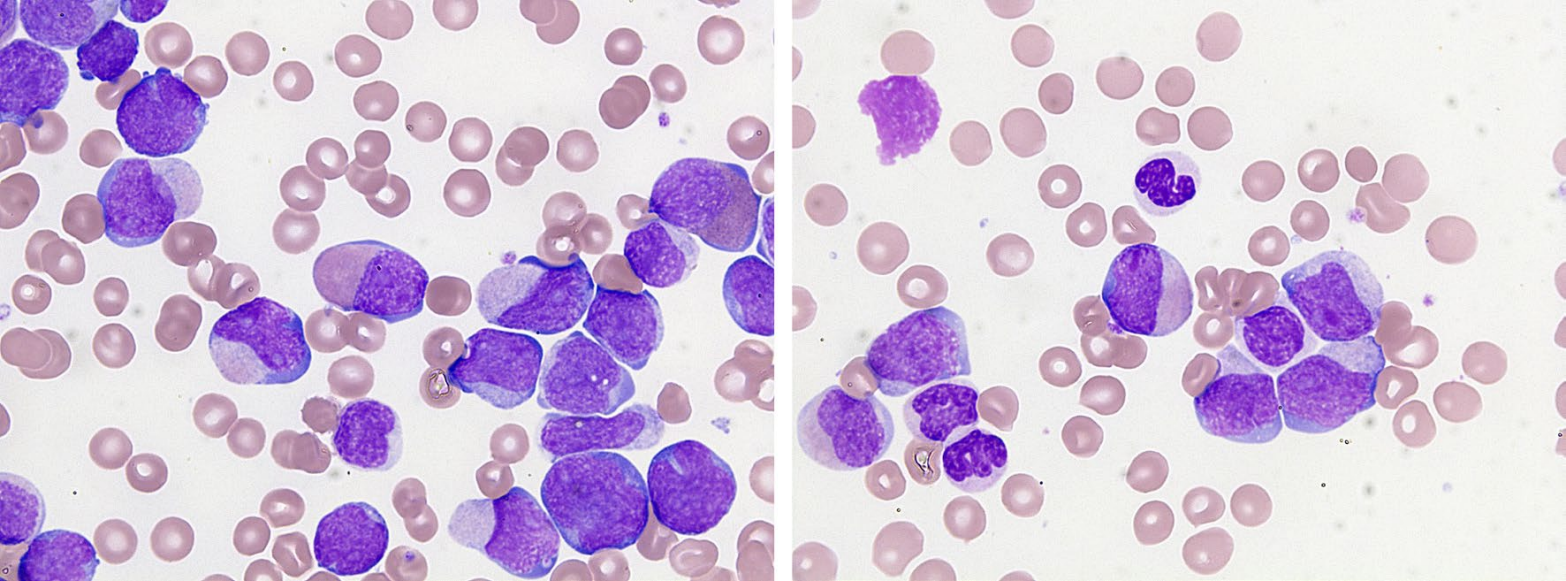

Morphology Update has recently discussed a patient with chronic myeloid leukemia (CML) presenting in blast crisis [1]. For comparison, we present here a patient with *BCR::ABL1*‐positive acute myeloid leukemia (AML). The patient was a 48‐year‐old man with no known history of CML, presenting with hepatomegaly and neurological abnormalities (headaches, agitation, and behavioral disturbance). His blood count showed hemoglobin concentration 64 g/L, white cell count 457.6 × 10^9^/L, and platelet count 31 × 10^9^/L. His blood film (images × 100 objective) showed 95% blast cells; these were large, with an intermediate nuclear‐cytoplasmic ratio, irregular nuclei, sometimes nucleoli, and cytoplasm containing atypical pink to lilac granules and occasional inclusions. Promyelocytes were present but myelocytes, eosinophils, and basophils were infrequent. Neutrophils were dysplastic with hypogranular cytoplasm and abnormal nuclear forms (right image). A bone marrow aspirate was hypercellular with 84% blast cells, without prominence of eosinophils or basophils. Cytogenetic analysis showed 46,XY,t(9;22)(q34;q11)[21]. A *BCR::ABL1* transcript, identified as BCR_E13::ABL1_E2, b2a2, p210, was detected on molecular analysis, together with mutations of *SMC3* and *WT1*. In view of the molecular findings and the lack of prominent basophilia, a diagnosis of *BCR::ABL1*‐positive AML appeared most likely but post‐treatment follow‐up was needed for confirmation.

The patient was treated with cytarabine, idarubicin, and imatinib as induction treatment (preceded by cytoreduction with hydroxycarbamide), followed by cytarabine and imatinib for consolidation. Additionally, cytarabine, methotrexate, and methylprednisolone were administered intrathecally to treat demonstrated central nervous system infiltration. Complete remission was achieved with the marrow being morphologically normal without neutrophilic, eosinophilic or basophilic hyperplasia. Cytogenetic analysis was normal at 2 months from diagnosis and a deep molecular remission was demonstrated at 4 months.

Diagnosis of *BCR::ABL1*‐positive AML requires the features of AML and demonstration of *BCR::ABL1*. However an essential diagnostic criterion is that there should be “a lack of features of CML before or at diagnosis and after therapy” [2]. The diagnosis is thus provisional until post‐treatment follow‐up excludes an alternative diagnosis of blastic presentation of CML. *De novo* AML cases have less frequent splenomegaly, a higher blast percentage and lower basophil numbers [2]. The precise *BCR::ABL1* transcript that is detected is not helpful because p210 is most often observed in both CML and *BCR::ABL1*‐positive AML [2].

## Conflicts of Interest

The authors declare no conflicts of interest.
